# Comprehensive contact tracing during an outbreak of alpha-variant SARS-CoV-2 in a rural community reveals less viral genomic diversity and higher household secondary attack rates than expected

**DOI:** 10.1128/msphere.00114-24

**Published:** 2024-08-07

**Authors:** Audun Sivertsen, Nicolay Mortensen, Unni Solem, Eivind Valen, Marie Francoise Bullita, Knut-Arne Wensaas, Sverre Litleskare, Guri Rørtveit, Harleen M. S. Grewal, Elling Ulvestad

**Affiliations:** 1 Department of Microbiology, Haukeland University Hospital, Bergen, Norway; 2 Ulvik municipality, Ulvik, Norway; 3 Computational Biology Unit, Department of Informatics, University of Bergen, Bergen, Norway; 4 NORCE Norwegian Research Centre, Research Unit for General Practice, Bergen, Norway; 5 Department of Global Public Health and Primary Care, University of Bergen, Bergen, Norway; 6 Department of Clinical Science, Bergen Integrated Diagnostic Stewardship Cluster, Faculty of Medicine, University of Bergen, Bergen, Norway; University of Michigan, Ann Arbor, Michigan, USA

**Keywords:** coronavirus, genome analysis, molecular epidemiology

## Abstract

Sequencing of severe acute respiratory syndrome coronavirus 2 (SARS-CoV-2) genomes throughout the COVID-19 pandemic has generated a wealth of data on viral evolution across populations, but only a few studies have so far explored SARS-CoV-2 evolution across large connected transmission networks. Here, we couple data from SARS-CoV-2 sequencing with contact tracing data from an outbreak with a single origin in a rural Norwegian community where samples from all exposed persons were collected prospectively. A total of 134 nasopharyngeal samples were positive by PCR. Among the 121 retrievable genomes, 81 were identical to the genome of the introductor, thus demonstrating that genomics beyond clustering genotypically similar viral genomes to confirm relatedness offers limited additional value to manual contact tracing. In the cases where mutations were discovered, five small genetic clusters were identified. We observed a household secondary attack rate of 77%, with 92% of household members infected among households with secondary transmission, suggesting that SARS-CoV-2 introduction into large families is likely to affect all household members.

**IMPORTANCE:**

In outbreak investigations, obtaining a full overview of infected individuals within a population is seldom achieved. We here present an example where a single introduction of B1.1.7 SARS-CoV-2 within a rural community allowed for tracing of the virus from an introductor via dissemination through larger gatherings into households. The outbreak occurred before widespread vaccination, allowing for a “natural” outbreak development with community lockdown. We show through sequencing that the virus can infect up to five consecutive persons without gaining mutations, thereby showing that contact tracing seems more important than sequencing for local outbreak investigations in settings with few alternative introductory transmission pathways. We also show how larger households where a child introduced transmission appeared more likely to promote further spread of the virus compared to households with an adult as the primary introductor.

## INTRODUCTION

Coronavirus infectious disease-2019 (COVID-19) is caused by the severe acute respiratory syndrome coronavirus 2 (SARS-CoV-2). The disease emerged in December 2019 in Wuhan, China, and was declared a pandemic by the World Health Organization on 11 March 2020. Several strategies, including manual tracing of contacts of patients with positive PCR results and investigations of viral genome variability in samples from patients infected with SARS-CoV-2, have been advocated to curb the spread and to track the routes of transmission during outbreaks (1–3).

Although important for restricting viral spread, the strategies are prone to miss asymptomatic patients who often remain untested, and patients who present with symptoms before viral shedding have reached the threshold for PCR detection (4). Furthermore, elucidation of the transmission chain has been hampered by the low mutation rate of SARS-CoV-2, the high frequency of identical genomes across several links of transmission, and variations in methods of sampling.

While retrospective studies identifying symptomatic cases report household secondary attack rate (HSAR) frequencies of 25%–50% (5), prospective sampling with symptomatic and asymptomatic cases exceeds 90% (4), especially in households with many residents (6). Transmission mapping limitations may be overcome by coupling data from viral genome variability to epidemiological investigations (7–10) and through modeling supported by large sets of metadata (11–13).

We here present a cluster of 134 persons infected with SARS-CoV-2 from a single origin in a relatively confined population. Using data from highly curated manual contact tracing, we compare transmission routes with the occurrence of mutations in the outbreak strain and also estimate the HSAR of the affected households.

## RESULTS

### Background and outbreak description

Ulvik is a municipality in western Norway with 1,080 inhabitants, 1 school, 1 kindergarten, and 1 nursing home. On 25 January 2021, Ulvik experienced an outbreak of SARS-CoV-2. Prior to the outbreak, only seven cases of SARS-CoV-2 had been detected and only institutionalized elderly patients had been vaccinated against COVID-19. The patterns of housing, congregations, and close encounters in rural Ulvik are well defined, thus facilitating contact tracing of high quality.

**Fig 1:**
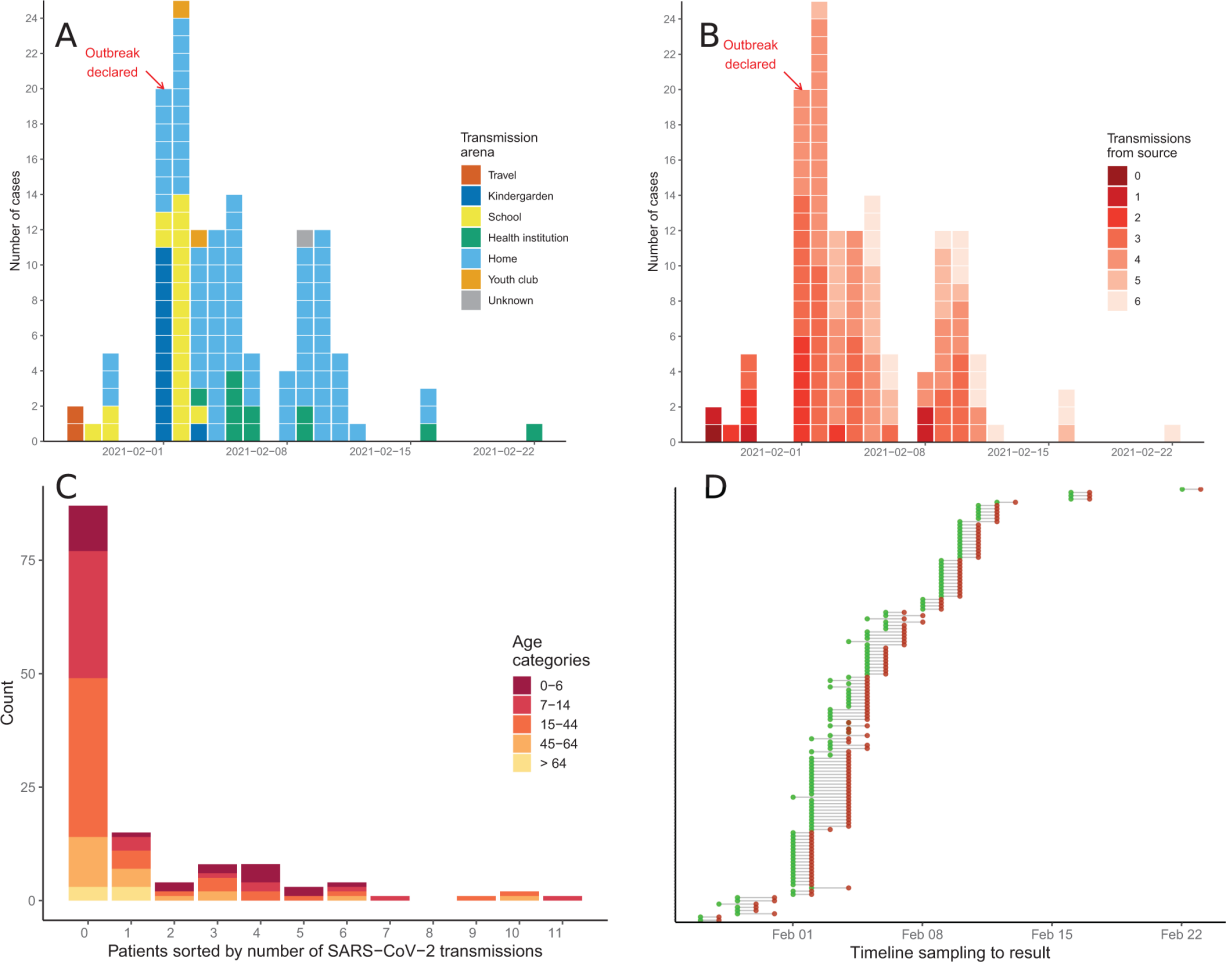
Epicurves and metadata. (**A**) Epidemiological curve showing arrival of samples to the regional microbiological laboratory on a timeline, colored by probable infection arena. (**B**) Same as panel A, colored by probable place in the transmission chain from source, judged by contact tracing. (C) Bar chart quantifying the number of patients who infect a given number of others, by extracting the number of outbound edges from all nodes in the contact tracing network. Bars are colored by age groups. (**D**) Lollipop chart showing dates of sampling (green dots) and feedback of a positive test result (red dot) for all persons in the data set.

**Fig 2:**
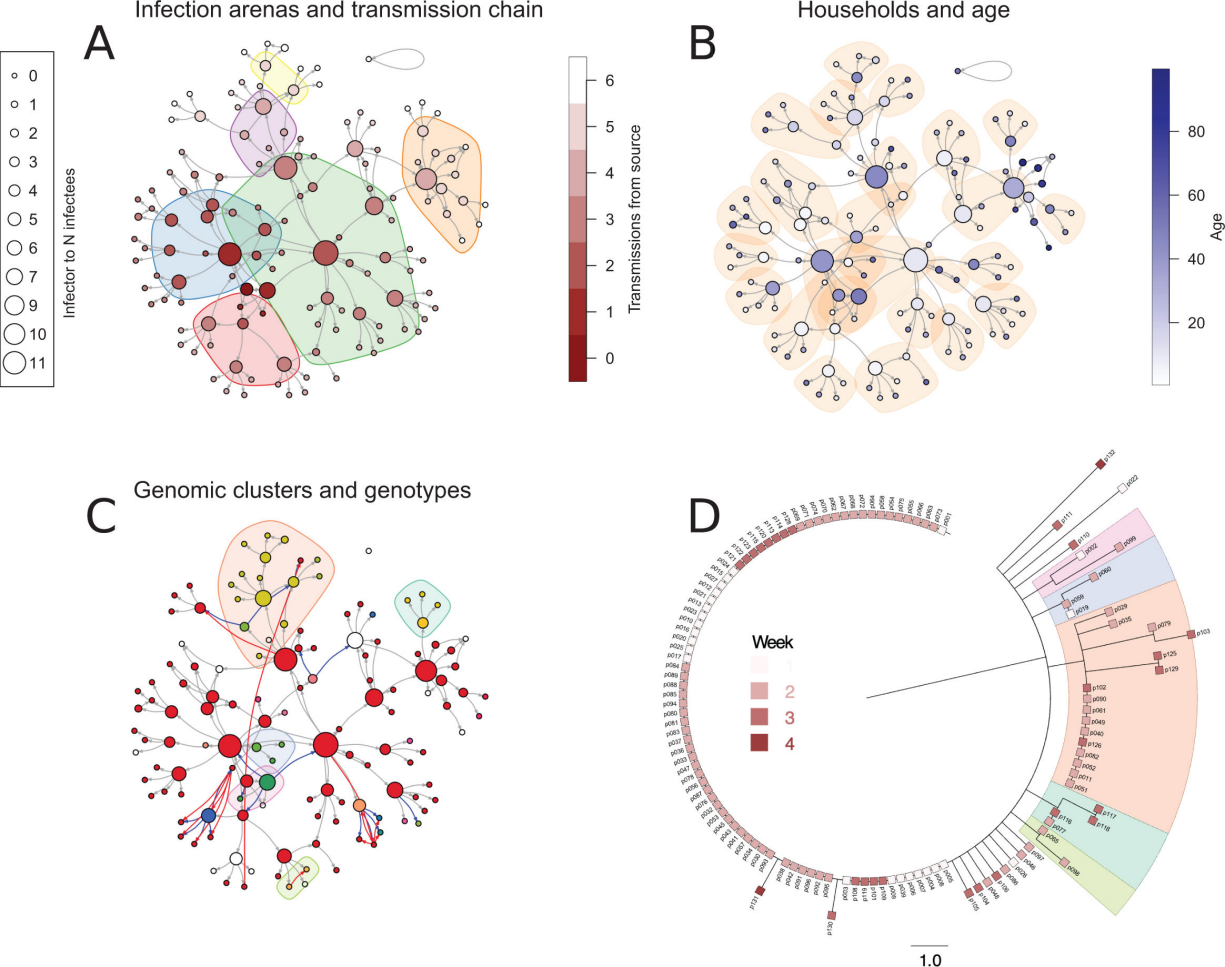
Transmission network connected with metadata and SARS-CoV-2 phylogeny. (A–C) Stylized transmission networks. Nodes represent persons within the network, and node size refers to the number of connected edges/transmissions. (A) Transmission across zones. The nodes are colored by place in the transmission chain. Transmission clusters outside households (two kindergarten zones [blue, red], two school classes [green, purple], a youth club [yellow], and an elderly care center [orange]) are highlighted in colored shades. (B) Transmission within households. Nodes are colored by shades of blue by age. Shades represent households. (C) Variant calling of virus genomes resulted in 28 distinct genotypes with one or more SNPs. Each node is colored with divergent colors representing each distinct genotype. The source strain genotype appears red. Phylogenetic clusters from common informative SNPs appear as colored shades. Blue edges represent transmissions made likely by manual tracing but unlikely given genomic data. Red edges are novel transmission links identified through retrospective interrogations of contacts between patients’ given genomic data. (D) Unrooted phylogenetic tree of all 121 available SARS-CoV-2 genomes as squares which are colored by sampling date from the first week (pale red) to the fourth week (dark red) of the outbreak. Underlying shades highlight genetic clusters corresponding to underlying shades in panel C. Scale shows the number of mutations from source.

As demonstrated in the epidemiological curve (Fig. 1A), two individuals introduced SARS-CoV-2 through travel abroad, of which one did not display symptoms at all and the other displayed symptoms after the 10th day of the obligatory quarantine period imposed on travelers from abroad. There was transmission within the family, which further led to dissemination within the school and kindergarten (Fig. 1A and 2A). SARS-CoV-2-positive test reports and contact tracing obtained on day 1 of the outbreak, Friday, 29 January 2021, showed that pupils from the school and kindergarten further transmitted SARS-CoV2 to their homes and a youth club. In one instance, a parent contracted SARS-CoV-2 and subsequently went to work in the nursing home, which led to a secondary outbreak there (Fig. 2A). All individuals in the kindergarten and in two school classes were quarantined. At day 4 of the outbreak (Monday, 1 February 2021), contact tracing suggested that 7 out of 10 school classes had been exposed to SARS-CoV-2. The local health authorities engaged in outbreak countermeasures and decided to close the school. The same day, the laboratory confirmed that the transmitting SARS-CoV-2 virus was an alpha variant (B1.1.7).

The chain of transmission was traced through six transmission links (Fig. 1B and 2A) as the municipal head physician and other health personnel kept track of who likely infected whom progressively as each infectee tested positive for SARS-CoV-2. This was done through contact tracing software as well as through mapping the likely transmission tree through a visualization on the wall of the municipal head physician’s office (Fig. S1). Contact tracing also included data on the most probable arena of infection (Fig. 2A), who lived with whom in households (Fig. 2B) as well as the age of the infected (Fig. 2B). A selection of metadata is presented in Table S1, and transmission links are presented in Table S2.

Nasopharyngeal sampling, transportation of samples to Haukeland University Hospital, PCR testing, and communication of test results to the health authorities in Ulvik were done within 1–2 days for most samples (Fig. 1D). A total of 809 samples for testing were obtained from 554 unique persons during the outbreak period, of which 134 persons were confirmed as SARS-CoV-2 positive by PCR. Twelve percent of the population was infected, a large proportion of whom were children and young adults.

### SARS-CoV-2 transmission dynamics

Figure 2A shows a stylized version of the transmission network derived from the comprehensive tracing of contacts, with transmission arenas outside households as colored shades underneath the network. Each node represents a case, and the size of the nodes reflects the number of connected edges. Edge direction shows likely transmission events based on contact tracing. Some persons may have several potential infectors. In this outbreak, four persons transmitted SARS-CoV-2 to nine or more persons, thereby acting as “superspreaders” in the transmission network (Fig. 1C, also corresponding to the four largest nodes in Fig. 2A through C). Three of these introduced SARS-CoV-2 in the kindergarten and school, in arenas with many potential infectees. One “superspreader” also worked at the nursing home, thereby causing an outbreak among residents and employees.

By adding metadata to the transmission network, we were able to identify the likely transmission routes within families as depicted in Fig. 2B, where underlying shades correspond to households. All nodes are colored in shades of blue by age with the oldest in the deepest shade. The age distribution of cases ranged from 2 to 99 years, but babies under the age of 2 were not tested due to the invasive nature of nasopharyngeal/throat swabs. Secondary transmission to begin with mostly occurred within households, where infectors had access to fewer infectees (Fig. 1D and 2B). Children (age 0–14) are not particularly over-represented as infectors who transmitted SARS-CoV-2 to one to seven infectees (Fig. 1C), but they are unlikely to live as single dwellers and thereby were drivers of secondary transmission within the community.

### SARS-CoV-2 sequences do not suffice to specify transmission events

All positive SARS-CoV-2 PCR tests were secured from routine disposal as samples are normally only stored for 1 week after testing and subsequently sequenced with Illumina or Oxford Nanopore either through the Swift Normalase Amplicon Panel (SNAP) or ARCTIC v2 protocol, respectively. After filtering out sequences with poor coverage, 121 SARS-CoV-2 genomes contained sufficient genetic information to be included in a phylogeny analysis by Nextstrain. Eighty one (67%) of the 121 viral genomes were identical to that of the source patient (hereafter called source strain to distinguish it from other isolates with mutations); see unrooted phylogeny in Fig. 2D and color of nodes in Fig. 2C. As depicted in Fig. 2C, the virus could pass through up to five transmissions without displaying mutations. Of the remaining genomes, some had shared SNPs, thereby enabling the identification of five clusters; see correspondingly colored underlying shades in the phylogenetic tree (Fig. 2D) and in the transmission network depicted in Fig. 2C.

A comparison of the manually curated transmission network and the network based on genomic information revealed some contradictory transmission routes. In all cases, but one, where the transmission arrow was transposed by the addition of genomic data, the change originated from a mutated genome being present within a transmission chain in which infectees carried the source strain. By performing local re-interrogations of contact points in these cases, some alternative transmission routes could be added (red edges, Fig. 2C), and others could be subtracted (blue edges, Fig. 2C).

Two families had a primary infector whose SARS-CoV-2 genome contained a mutation that did not propagate to other household members, of which several had the source strain, thus necessitating the identification of alternative household index patients.

In another case, a child did not receive the SARS-CoV-2 virus through household transmission of a mutated variant but instead received the source strain from attendance in kindergarten. In a third case, a previously undisclosed longer conversation led to transmission of the source strain, where the earlier presumed transmitter carried a mutated strain, which was unlikely to revert back to the source strain in the infectee.

In a fourth case, a cluster arose within a single family, where the infector had the source strain and three of four infectees carried strains with mutations. Two of the three mutated strains shared a common SNP. It is unclear whether the two connected mutated strains represented independent transmission between these two family members or if the SARS-CoV-2 strain of the household index patient had hypermutability throughout the infective period.

The only unresolved discrepancy between epidemiological and genomic data was a single strain (Fig. 2C, blue node with inbound blue edge at the lower left side) where genomic data pointed to inclusion within the top cluster (orange shade, Fig. 2C), but no epidemiological connection could be found. This particular SARS-CoV-2 genome suffered from relatively poor coverage (median 652) and alignment to the reference strain (82.8%), and we cannot exclude base-calling errors as a confounding factor.

### Dense sampling gives a household secondary attack rate of 77%

In addition to the 130 infectees who have a known family constellation (4 institutionalized patients or single dwellers were not included) (Fig. 2B), 25 additional household members did not test positive for SARS-CoV-2 despite performing repeated nasopharyngeal swabs for PCR analysis (Table S3) and are not shown in the network.

**Fig 3:**
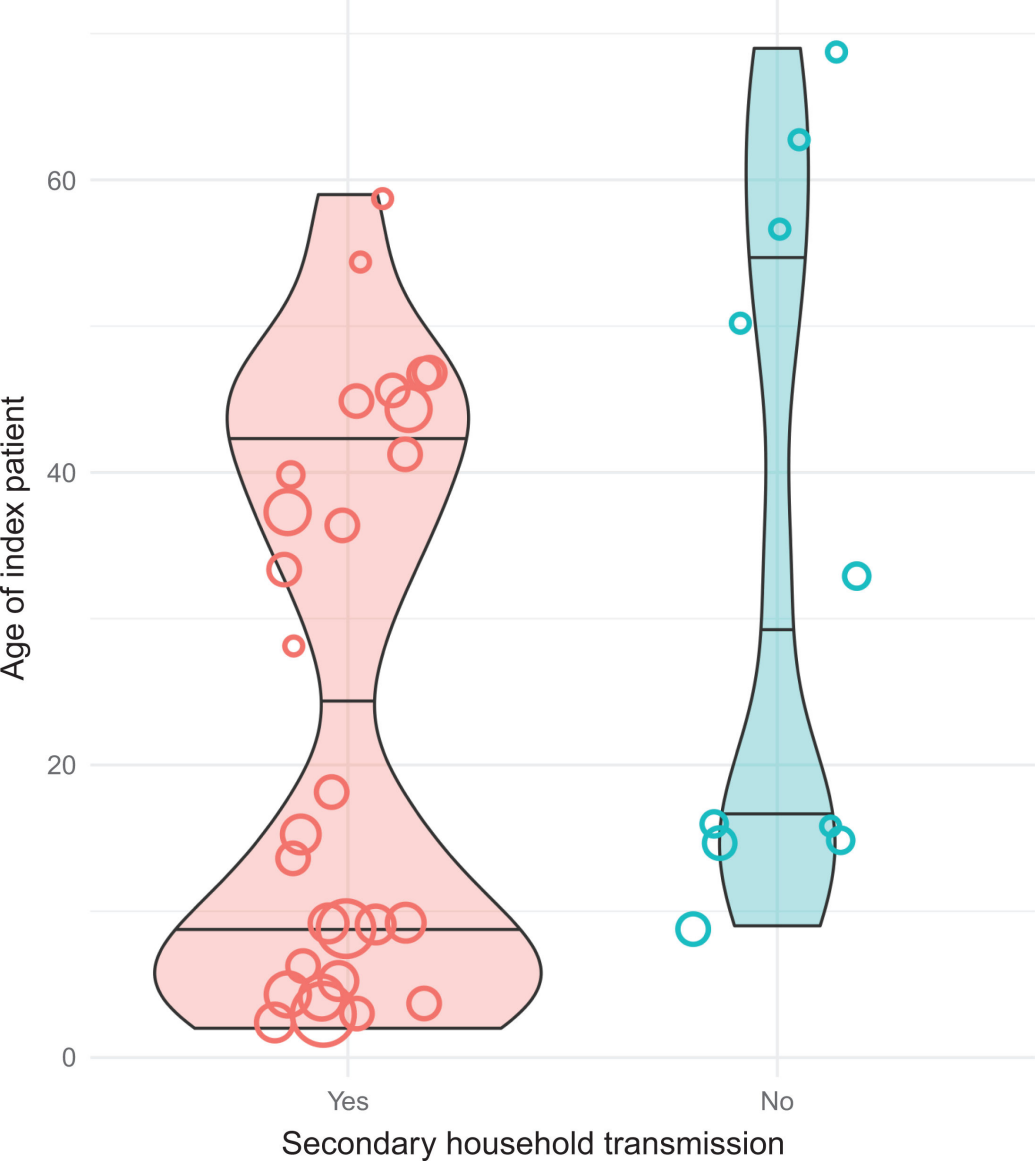
Household index patient’s age (*y*-axis) in households with and without secondary transmission (*x*-axis). Each circle represents a household, and width represents the household size. Violin plots show the age distribution, in which violin width is normalized to count and reflects the number of households with household index patients in the given age range.

Thirty-eight households in the network consist of at least 2 persons, with a total of 155 residents. Of these households, 74% (28/38) experienced secondary transmissions. Assuming a single household index case per household (38 in total), the secondary attack rate in this population is 77% (92/117). Limiting the denominator to include only members of households with secondary transmission, the secondary transmission rate is 92% (92/100). This indicates the extent of secondary transmission if a household was not able to contain its index case. The feasibility of containment measures is further demonstrated by examining the 13 households where the household index case is a child. Only 1/13 of these households (8%) was able to limit further spread of the virus, as opposed to 9/25 (36%) households with an adolescent (>13 years) or older as the household index case (*P* = 0.118). Figure 3 illustrates how households with secondary transmission more often appear to have household index cases in the lower end of the age spectrum. The size of the dots reflects the number of people in each household. Those households where secondary transmission did not take place were characterized by being smaller (two to three persons) and consisted of adults living with other adults and/or with teenagers.

## DISCUSSION

We present the dynamics of a large connected outbreak of COVID-19 caused by the SARS-CoV-2 B.1.1.7 strain. The outbreak occurred in a local community, thus demarcating it from previously reported densely sampled outbreaks occurring in healthcare institutions (11, 13). The ecological settings and transmission dynamics are therefore distinct, as contact between households is likely less prevalent than contact between rooms in a ward or between wards in a health institution. The outbreak occurred before population-wide vaccination took place, thus providing a glimpse of SARS-CoV-2 propagation throughout an immunologically naïve population.

Of the 134 PCR-positive samples, 121 sequences could be extracted to create consensus sequences. All sequences clustered well together, confirming that this outbreak was of a single origin. Of 121 genomes 81 were identical, and these identical sequences could be retrieved through as many as five transmission links across six individuals. This underscores that genomics does not provide enough resolution to resolve SARS-CoV-2 transmission between infector and infectee on such a granular scale as displayed here or by others (8, 10, 13, 14), in contexts of outbreaks with a single origin. We chose to abstain from calculating minor variants as they seem to exist between a minority of transmission pair SARS-CoV-2 genomes, have limited ability to inform transmission directionality, and apparently most often arise *de novo* within infected patients (15–17). Given the mutation frequency of SARS-CoV-2 compared to other viral pathogens, which is not exceptionally high (18), we presume that viral genomics in densely sampled outbreaks, in general, may show similar limited utility with transmission chain resolution compared to epidemiological data.

Our sequencing results are consistent with results obtained during a large community outbreak of the Delta (B.1.617) variant in China (*n* = 167) (10). A similar absence of genetic variance between samples was found, with most SARS-CoV-2 sequences being identical with occasional clusters of isolates with singular or common SNPs. However, the study did not report the vaccination status of infected persons nor the HSAR, thus limiting the results for resolving the transmission dynamics in naïve populations.

We observe a high HSAR compared to other estimates (5). There are several reasons why we believe we were able to obtain accurate estimates of HSAR in this outbreak. First, the calculation of HSAR relies on identifying all cases in an exposed population, regardless of symptom presence or varying incubation time across infected individuals. As such, infrequent, symptom-based or delayed sampling could limit case detection when utilizing PCR. Retrospective case identification by serology fails to identify all cases (19). Second, in contrast to others (20), we did not exclude households where several persons may have introduced SARS-CoV-2 into the household, as this occurred in a minority of cases. Third, as this is a single outbreak in a specific context, parts of the difference may be explained by demographical differences.

As this community is interconnected, contagion could emanate from several sources, as corrected transmission pathways in Fig. 2C show. As possible hidden “cross-transmissions” are likely to occur also in other data sets and are hard to correct without accompanying metadata, we still believe that HSAR rates calculated here are indicative of real-life transmission dynamics in different types of households.

Multiple circulating lineages introduced into the community at once may disrupt contact tracing and assumptions of transmission directionality. Iterative progress in design of HSAR studies during the continuation of the pandemic may have resulted in better capture of infected individuals, thus downplaying secondary attack rate in early variants. In population surveillance settings, identifying the denominator would be difficult, as identifying the number of exposed persons is uncertain for many COVID-19 cases.

Although children were the drivers of transmission in this outbreak by accounting for a larger proportion of people infected than adults, three out of the four “superspreaders” who transmitted SARS-CoV-2 to many others were adults and likely reflect that adults more often than children move between different social arenas in the network.

We observed several instances of successful household isolation where no other household members got infected, particularly in households with few persons and adolescent or adult household index persons. In contrast, households with many members and children were not able to contain transmission within the household. Although the majority of the drivers of infection within households were children or caregivers of children in this outbreak, we do not have enough data points to suggest that a child as the index patient enables transmission of SARS-CoV-2 within households to a larger extent compared to households with adolescents or adult index patients. A meta-analysis comparing SARS-CoV-2 transmission from adults and children found similar secondary transmission rates of SARS-CoV-2 among the groups (21). The transmission dynamics may then be explained by the demography of the population, with many large households with small children who cannot be physically separated from their families. Households with secondary transmission often have many members, which include children (as shown in Fig. 3). Therefore, the exclusion of households with potentially more than one primary infector will bias results toward lower HSAR.

Some limitations exist—the capture of asymptomatic cases is still an issue, and during the outbreak, no standardized research protocol for sampling exposed individuals was in place. Variability in testing compliance, especially when testing at multiple time points, is to be expected. However, testing all exposed persons at least once at a time point where the likelihood of obtaining a positive test is high, in addition to good compliance for testing in persons with either symptoms or possible exposure, suggests that most infected cases were discovered. Hidden transmission would likely cause symptomatic disease locally outside known paths of exposure—only one positive test originated from an asymptomatic person without a known link to any infector.

In conclusion, the data gathered by this single outbreak show that genomic sequencing of SARS-CoV-2 is not likely to inform transmission events in already connected clusters but may occasionally provide extra data when mutations occur. During an outbreak, HSAR rates are affected by how many people are living within the household. If children introduced SARS-CoV-2 into a household, transmission often involved all other household members.

## MATERIALS AND METHODS

### Contact tracing and SARS-CoV-2 testing

After the source patient had been identified, it was assumed that any positive cases from then on would be epidemiologically linked as there were no prior positive tests that month in this municipality, and exposed persons were quickly quarantined. A team consisting of personnel from the local doctors office and other health care workers kept overview of the contact tracing effort through a contact tracing map on the wall of the municipal head physician (Fig. S1, picture has also been published in a local newspaper). They also used a proprietary contact tracing software (www.remin.no, ReMin A/S, Nå, Norway) for the collection of patient metadata, including place of exposure, household members in each affected household, allocation of isolation or quarantining of the infected and the exposed, and age and gender. SARS-CoV-2 testing was physically performed by visiting each household with quarantined non-institutionalized persons and PCR testing regardless of symptoms, with revisits for successive testing of other household members as the index case received a positive test, again regardless of symptoms. The same health personnel team also treated patients who obtained complications arising from the infection and also conducted hospital referrals when needed.

### SARS-CoV-2 RNA extraction and PCR

Nucleic acids from the nasopharyngeal sample were extracted on the MagnaPure96 platform (Roche, Mannheim, Germany) using the MagNA Pure 96 DNA and Viral NA Large Volume Kit (Roche). The in-house real-time PCR was based on the E-gene primers and probes as described by Corman et al. (22) and was run on a LightCycler 480 instrument (Roche) in a 20-µL reaction volume using the QuantiNova Pathogen MasterMix (Qiagen, Hilden, Germany). Ct values for positive samples are given in Table S1.

### SARS-CoV-2 RNA sequencing

Viral RNAs from 127 of 134 total samples were sequenced by the Illumina NovaSeq 6000 (Illumina, San Diego) with 150-bp paired-end reads. The library was generated by SNAP SARS-CoV-2 with additional genome coverage. The pipeline used for sequence quality assessment, variant calling, and consensus sequence generation is available at https://github.com/nsc-norway/covid-seq. Four genomes were sequenced at the Norwegian Institute of Public Health using the Oxford Nanopore GridION (Oxford Nanopore Technologies, Oxford), with preceding sample preparation using the ARTIC protocol v.2 (23). Variant calling and consensus sequence generation of Illumina sequences were done with iVar (24). The phylogeny from nextstrain.org (25) was used to define clusters. Three samples were lost during sample retrieval. All sequence reads are available in ENA BioProject PRJEB65109, with individual sample accessions provided in Table S1.

### Household secondary attack rates

The HSAR was estimated by dividing the number of infected household members (not counting the person introducing the infection to the household) by the total number of household members eligible to be infected by the household index person. Fisher’s exact test was used to compare the proportion of households with secondary transmission and a child as the primary infector as opposed to an adult. A child in this setting was defined as a person below 13 years.

### Computational analyses

Figures and network plots were made in R, using iGraph, GGally, RColorBrewer, EpiCurve, and ggplot2 packages (26–28).

## Data Availability

All sequence reads are available in ENA BioProject PRJEB65109, with individual sample accessions provided in Table S1.

## SUPPLEMENTAL MATERIAL

Figure S1 msphere.00114-24-s0001.pdf Contact tracing network.

Table S1 msphere.00114-24-s0002.xlsx Metadata of patients, PCR, and sequencing.

Table S2 msphere.00114-24-s0003.xlsx Contact tracing data.

Table S3 msphere.00114-24-s0004.xlsx Household secondary transmission data.

ASM does not own the copyrights to Supplemental Material that may be linked to, or accessed through, an article. The authors have granted ASM a non-exclusive, world-wide license to publish the Supplemental Material files. Please contact the corresponding author directly for reuse.

## References

[1] Rockett RJ, Arnott A, Lam C, Sadsad R, Timms V, Gray K-A, Eden J-S, Chang S, Gall M, Draper J, Sim EM, Bachmann NL, Carter I, Basile K, Byun R, O’Sullivan MV, Chen SC-A, Maddocks S, Sorrell TC, Dwyer DE, Holmes EC, Kok J, Prokopenko M, Sintchenko V. 2020. Revealing COVID-19 transmission in Australia by SARS-CoV-2 genome sequencing and agent-based modeling. Nat Med 26:1398–1404. doi:10.1038/s41591-020-1000-7 32647358

[2] Hjorleifsson KE, Rognvaldsson S, Jonsson H, Agustsdottir AB, Andresdottir M, Birgisdottir K, Eiriksson O, Eythorsson ES, Fridriksdottir R, Georgsson G, et al.. 2022. Reconstruction of a large-scale outbreak of SARS-CoV-2 infection in Iceland informs vaccination strategies. Clin Microbiol Infect 28:852–858. doi:10.1016/j.cmi.2022.02.012 35182757 PMC8849849

[3] Lemieux JE, Siddle KJ, Shaw BM, Loreth C, Schaffner SF, Gladden-Young A, Adams G, Fink T, Tomkins-Tinch CH, Krasilnikova LA, et al.. 2021. Phylogenetic analysis of SARS-CoV-2 in Boston highlights the impact of superspreading events. Science 371:eabe3261. doi:10.1126/science.abe3261 33303686 PMC7857412

[4] Rasmussen AL, Popescu SV. 2021. SARS-CoV-2 transmission without symptoms. Science 371:1206–1207. doi:10.1126/science.abf9569 33737476

[5] Madewell ZJ, Yang Y, Longini IM, Halloran ME, Dean NE. 2022. Household secondary attack rates of SARS-CoV-2 by variant and vaccination status. JAMA Netw Open 5:e229317. doi:10.1001/jamanetworkopen.2022.9317 35482308 PMC9051991

[6] Cerami C, Rapp T, Lin F-C, Tompkins K, Basham C, Muller MS, Whittelsey M, Zhang H, Chhetri SB, Smith J, Litel C, Lin K, Churiwal M, Khan S, Claman F, Rubinstein R, Mollan K, Wohl D, Premkumar L, Juliano JJ, Lin JT. 2021. High household transmission of SARS-CoV-2 in the United States: living density, viral load, and disproportionate impact on communities of color. Clin infect DIS. doi:10.1101/2021.03.10.21253173 PMC8436395 34383889

[7] Hamilton WL, Tonkin-Hill G, Smith ER, Aggarwal D, Houldcroft CJ, Warne B, Meredith LW, Hosmillo M, Jahun AS, Curran MD, et al.. 2021. Genomic epidemiology of COVID-19 in care homes in the east of England. Elife 10:e64618. doi:10.7554/eLife.64618 33650490 PMC7997667

[8] Popa A, Genger J-W, Nicholson MD, Penz T, Schmid D, Aberle SW, Agerer B, Lercher A, Endler L, Colaço H, et al.. 2020. Genomic epidemiology of superspreading events in Austria reveals mutational dynamics and transmission properties of SARS-CoV-2. Sci Transl Med 12:eabe2555. doi:10.1126/scitranslmed.abe2555 33229462 PMC7857414

[9] Løvestad AH, Jørgensen SB, Handal N, Ambur OH, Aamot HV. 2021. Investigation of intra-hospital SARS-CoV-2 transmission using nanopore whole-genome sequencing. J Hosp Infect 111:107–116. doi:10.1016/j.jhin.2021.02.022 33647375 PMC7908852

[10] Li B, Deng A, Li K, Hu Y, Li Z, Shi Y, Xiong Q, Liu Z, Guo Q, Zou L, et al.. 2022. Viral infection and transmission in a large, well-traced outbreak caused by the SARS-CoV-2 Delta variant. Nat Commun 13:460. doi:10.1038/s41467-022-28089-y 35075154 PMC8786931

[11] Lindsey BB, Villabona-Arenas CJ, Campbell F, Keeley AJ, Parker MD, Shah DR, Parsons H, Zhang P, Kakkar N, Gallis M, et al.. 2022. Characterising within-hospital SARS-CoV-2 transmission events using epidemiological and viral genomic data across two pandemic waves. Nat Commun 13:671. doi:10.1038/s41467-022-28291-y 35115517 PMC8814040

[12] Campbell F, Didelot X, Fitzjohn R, Ferguson N, Cori A, Jombart T. 2018. outbreaker2: a modular platform for outbreak reconstruction. BMC Bioinformatics 19:363. doi:10.1186/s12859-018-2330-z 30343663 PMC6196407

[13] Abbas M, Robalo Nunes T, Cori A, Cordey S, Laubscher F, Baggio S, Jombart T, Iten A, Vieux L, Teixeira D, Perez M, Pittet D, Frangos E, Graf CE, Zingg W, Harbarth S. 2021. Explosive nosocomial outbreak of SARS-CoV-2 in a rehabilitation clinic: the limits of genomics for outbreak reconstruction. J Hosp Infect 117:124–134. doi:10.1016/j.jhin.2021.07.013 34461177 PMC8393517

[14] Gallego-García P, Varela N, Estévez-Gómez N, De Chiara L, Fernández-Silva I, Valverde D, Sapoval N, Treangen TJ, Regueiro B, Cabrera-Alvargonzález JJ, Del Campo V, Pérez S, Posada D. 2022. Limited genomic reconstruction of SARS-CoV-2 transmission history within local epidemiological clusters. Virus Evol 8:veac008. doi:10.1093/ve/veac008 35242361 PMC8889950

[15] Lythgoe KA, Hall M, Ferretti L, de Cesare M, MacIntyre-Cockett G, Trebes A, Andersson M, Otecko N, Wise EL, Moore N, et al.. 2021. SARS-CoV-2 within-host diversity and transmission. Science 372:eabg0821. doi:10.1126/science.abg0821 33688063 PMC8128293

[16] Tonkin-Hill G, Martincorena I, Amato R, Lawson ARJ, Gerstung M, Johnston I, Jackson DK, Park N, Lensing SV, Quail MA, et al.. 2021. Patterns of within-host genetic diversity in SARS-CoV-2. Elife 10:e66857. doi:10.7554/eLife.66857 34387545 PMC8363274

[17] Martin MA, Koelle K. 2021. Comment on “Genomic epidemiology of superspreading events in Austria reveals mutational dynamics and transmission properties of SARS-CoV-2”. Sci Transl Med 13:eabh1803. doi:10.1126/scitranslmed.abh1803 34705523 PMC9301528

[18] Markov PV, Ghafari M, Beer M, Lythgoe K, Simmonds P, Stilianakis NI, Katzourakis A. 2023. The evolution of SARS-CoV-2. Nat Rev Microbiol 21:361–379. doi:10.1038/s41579-023-00878-2 37020110

[19] Accorsi EK, Qiu X, Rumpler E, Kennedy-Shaffer L, Kahn R, Joshi K, Goldstein E, Stensrud MJ, Niehus R, Cevik M, Lipsitch M. 2021. How to detect and reduce potential sources of biases in studies of SARS-CoV-2 and COVID-19. Eur J Epidemiol 36:179–196. doi:10.1007/s10654-021-00727-7 33634345 PMC7906244

[20] Julin CH, Robertson AH, Hungnes O, Tunheim G, Bekkevold T, Laake I, Aune IF, Killengreen MF, Strand TR, Rykkvin R, Dorenberg DH, Stene-Johansen K, Berg ES, Bodin JE, Oftung F, Steens A, Næss LM. 2021. Household transmission of SARS-CoV-2: a prospective longitudinal study showing higher viral load and increased transmissibility of the alpha variant compared to previous strains. Microorganisms 9:2371. doi:10.3390/microorganisms9112371 34835495 PMC8622435

[21] Zhu Y, Xia Y, Pickering J, Bowen AC, Short KR. 2023. The role of children in transmission of SARS-CoV-2 variants of concern within households: an updated systematic review and meta-analysis, as at 30 June 2022. Euro Surveill 28:2200624. doi:10.2807/1560-7917.ES.2023.28.18.2200624 37140450 PMC10161681

[22] Corman VM, Landt O, Kaiser M, Molenkamp R, Meijer A, Chu DK, Bleicker T, Brünink S, Schneider J, Schmidt ML, Mulders DG, Haagmans BL, van der Veer B, van den Brink S, Wijsman L, Goderski G, Romette J-L, Ellis J, Zambon M, Peiris M, Goossens H, Reusken C, Koopmans MP, Drosten C. 2020. Detection of 2019 novel coronavirus (2019-nCoV) by real-time RT-PCR. Euro Surveill 25:2000045. doi:10.2807/1560-7917.ES.2020.25.3.2000045 31992387 PMC6988269

[23] Tyson JR, James P, Stoddart D, Sparks N, Wickenhagen A, Hall G, Choi JH, Lapointe H, Kamelian K, Smith AD, Prystajecky N, Goodfellow I, Wilson SJ, Harrigan R, Snutch TP, Loman NJ, Quick J. 2020. Improvements to the ARTIC multiplex PCR method for SARS-CoV-2 genome sequencing using nanopore. Genomics. doi:10.1101/2020.09.04.283077

[24] Grubaugh ND, Gangavarapu K, Quick J, Matteson NL, De Jesus JG, Main BJ, Tan AL, Paul LM, Brackney DE, Grewal S, Gurfield N, Van Rompay KKA, Isern S, Michael SF, Coffey LL, Loman NJ, Andersen KG. 2019. An amplicon-based sequencing framework for accurately measuring intrahost virus diversity using PrimalSeq and iVar. Genome Biol 20:8. doi:10.1186/s13059-018-1618-7 30621750 PMC6325816

[25] Hadfield J, Megill C, Bell SM, Huddleston J, Potter B, Callender C, Sagulenko P, Bedford T, Neher RA. 2018. Nextstrain: real-time tracking of pathogen evolution. Bioinformatics 34:4121–4123. doi:10.1093/bioinformatics/bty407 29790939 PMC6247931

[26] Schloerke B, Cook D, Larmarange J, Briatte F, Marbach M, Thoen E, Elberg A, Toomet O, Crowley J, Hofmann H, Wickham H, Toomet O, Crowley J, Hofmann H, Wickham H. 2022. GGally: extension to “ggplot2” (2.2.0). Available from: https://ggobi.github.io/ggally/, https://github.com/ggobi/ggally

[27] R Core Team. 2022. R: a language and environment for statistical computing. R Foundation for Statistical Computing, Vienna, Austria. https://www.R-project.org/.

[28] Csardi G, Nepusz T. 2006. The Igraph software package for complex network research. InterJ Complex Systems:1695.

