# Supplementary material for: Comprehensive contact tracing during an outbreak of alpha-variant SARS-CoV-2 in a rural community reveals less viral genomic diversity and higher household secondary attack rates than expected
Source: mSphere. 2024 Aug 7;9(8):e00114-24. doi: 10.1128/msphere.00114-24 (PMC11351100; doi:10.1128/msphere.00114-24)
Supplement: Figure S1 — Contact tracing network. [file msphere.00114-24-s0001.pdf]

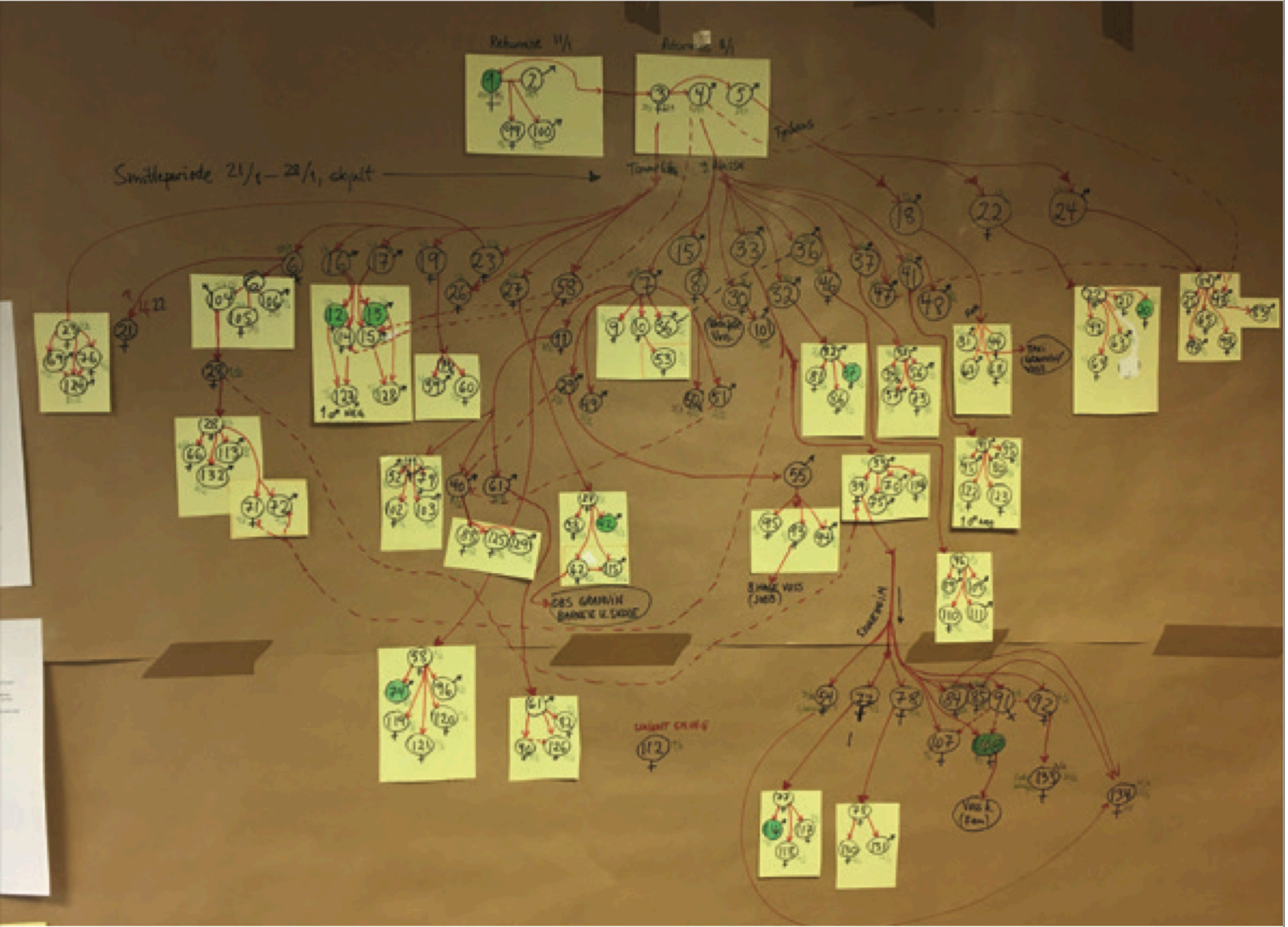

Figure S1: Contact tracing network. Picture of the office of the Municipal Head Physicians wall right after the outbreak. Reprinted with permission from the regional community medicine journal Utposten (https://www.utposten.no/).
